# The Moderating Roles of Resilience and Coping Strategy on Well-Being of Victimized Forensic Workers

**DOI:** 10.1177/0306624X221124834

**Published:** 2022-09-30

**Authors:** Caroline Rou, Marija Janković, Stefan Bogaerts

**Affiliations:** 1 Tilburg University, The Netherlands; 2 Fivoor Science and Treatment Innovation (FARID), Rotterdam, The Netherlands

**Keywords:** victimization, forensic workers, resilience, active coping, passive coping, avoidant coping

## Abstract

Previous research on workplace victimization has often disregarded forensic psychiatric populations and not yet been extended to the coronavirus pandemic. The present study expected the isolation of the government-issued lockdown to increase aggressive behavior in forensic patients, ultimately decreasing the general well-being of victimized forensic workers. Possible buffering protective factors (resilience and active coping) and enhancing risk factors (avoidant coping and passive coping) were investigated with the intention of optimizing the general well-being of at-risk forensic workers. The valid sample (*N* = 311) consisted of Dutch and Belgian forensic workers (74.6% females) with at least 9 hours of weekly patient contact, and with a mean age of 37.99 (*SD* = 12.20). Participants reported the number of violent incidents in the past 2 months, as well as completed a questionnaire battery including measures of well-being, resilience, and coping strategies. A significant increase of victimization during the lockdown compared to after it was lifted was found, however, the study did not find evidence to support that this negatively influenced the worker’s general well-being. Active coping was found to be a significant moderator and protective factor for the general well-being of victimized forensic workers. In contrast, resilience, avoidant and passive coping were not significant moderators in this association. The present study has valuable clinical implications that could lead to preparatory and preventative measures for forensic workers at risk of being victimized. Future research may investigate constructs such as life satisfaction and post-traumatic growth, as well as be broadened into prison populations.

The coronavirus disease (COVID-19) outbreak has caused more than 3.8 million deaths worldwide, according to researchers at Johns Hopkins University (Dong et al., 2020). In an effort to stagnate the transmission of the deadly disease, governmental restrictive measures were enacted, such as personal quarantine, minimized social gatherings, and strict social distancing rules. Although COVID-19 has had major economic, social, health, and environmental consequences (Nicola et al., 2020), the psychological well-being of individuals has also been severely affected. It has been shown that approximately 81% of young people showed signs of severe distress after being in an isolated lockdown for three weeks (Dangi et al., 2020). Other studies also show a robust association between freedom-restricting measures and post-traumatic stress, anxiety, and depressive symptoms (Guessoum et al., 2020; Mowbray, 2020). However, less is known about how the consequences of these measures on social and interpersonal intercourse affect individuals who are incarcerated in prison or closed secure mental health institutions as well as staff members who are in direct contact with them.

Before the pandemic, forensic inpatients living in closed secure mental health institutions had the opportunity to receive visits from friends and family as a form of social support. In addition, they could make use of leave modalities, such as unguided leaves as a crucial part of their resocialization (Walker et al., 2013). However, the lockdown measures stripped the opportunity for inpatients to receive external social support and all leave modalities, at least in the Netherlands, were suspended between March and the end of June 2020, which may buffer behavioral rehabilitation due to potential stress from lack of support (Coyne & Downey, 1991). The need for proper reintegration is important. Recent research suggests that the adverse effects of social isolation may (amongst others) worsen pre-existing symptoms of mental disorders and provoke aggressive behaviors (Torales et al., 2020). This finding is supported by additional evidence suggesting that experiencing a secondary lockdown during confinement may evoke an unwanted change in social cognition and behavior that could have previously been buffered by social support (Cohen, 2004).

Additional evidence suggests that the effects of the lockdown appear to have increased domestic abuse and child violence in forensic outpatients, proposing that aggressive inhibitions may propagate following the lockdown (Fovet et al., 2020). Direct repercussions of social isolation include symptoms such as accelerated cognitive decline, impaired executive function, and poor sleep quality (Novotney, 2020). There is substantial evidence that sleep dysregulation can increase the risk of behavioral problems and violent behavior. Previous research on inmate recidivism rates indicates that those who maintained a strong social network throughout confinement and were visited regularly were less likely to re-offend any crime than those who lacked any social support (Cochran, 2014).

In addition, there is an abundance of evidence suggesting that social support may serve as a buffer between major life changes for forensic patients, which in this case is isolation and psychological distress (Wilcox, 1981). These findings provide insight into the importance of social support for forensic patients, and that the confiscation of this valuable time can lead to enhancement of psychopathology and aggressive behavior. It might be that the lack of social support during lockdown measures increases aggressive behavior in forensic psychiatric patients. However, as far as is known, no previous research has investigated the influence of the lockdown measures on institutional aggression in forensic psychiatric inpatients.

If violence in forensic psychiatric facilities has increased since the lockdown, then forensic workers might be at an elevated risk of being victimized. Institutional victimization can be defined as being subjected to flagrant acts, in the form of either physical or verbal misconduct, which ultimately leads the sufferer of the directed behavior to be compromised (Hartjen & Priyadarsini, 2012). It is not uncustomary for psychiatric staff to experience assault or aggression in the workplace from inmates or patients. A recent study by Kelly et al. (2016) regarding the well-being and safety among psychiatric staff in inpatient facilities investigated the harmful impact of conflict and assault at the workplace. Evident consequences include the reduction in emotional and physical health, such as elevated shock, anxiety, anger, and irritability (Needham et al., 2005). Research has also indicated that exposure to victimizing events in the workplace can put psychiatric staff at an increased risk of developing post-traumatic stress and depressive symptomatology (Richter & Berger, 2006). Further evidence suggests that the severity of post-traumatic symptoms and the kind of violence workers are exposed to, verbal or physical, may be contingent on personality attributes (Jankovic et al., 2021). Acts of physical assault toward staff can also have physical consequences, such as severe headaches and tension, or even permanent injury (Gerberich et al., 2004).

Alongside severe emotional and possible physical trauma of victimization, aggression toward psychiatric staff has also been found to be significantly associated with occupational stress, ultimately compromising job satisfaction and self-efficacy of workers (Yao et al., 2014). There is also a clear association between accounts of inpatient aggression and the number of days of leave from work, showing that physical violence by inpatients is the strongest predictor of psychiatric employees calling in sick (Nijman et al., 2005). Beyond work dissatisfaction, victimization has a critical influence on the broader scale of general well-being. General well-being is conceptualized by the presence of positive emotions, the overarching absence of negative emotions, and living an overall fulfilled life (Gaston & Vogl, 2005). Although most studies have been conducted in general psychiatric facilities, it is likely that forensic workers experience even more victimization than workers in general mental health institutions, due to previous patient history of crime or aggression (Jankovic et al., 2021)

Due to the rehabilitation process being enduring and strenuous for the forensic inpatients, it may be refractory to solely rely on confronting the elemental core of the violent behavior as the means to reduce it. Although the decrease of aggressive behavior should be pivotal, there may be internal protective traits of the workers that can buffer the negative effects of workplace victimization on general well-being (Jankovicet al., 2021 ). There are individual differences in how individuals recover in the aftermath of adversity. In the context of criminal victimization, some forensic workers may plummet into a downward spiral of pervasive ramifications, such as polysubstance abuse (Charak et al., 2015), permanent psychological dysfunction, or poor social adjustment (Wayland et al., 1991). Contrastingly, some individuals may emotionally ricochet after such an incident as if nothing, in particular, had happened, and this effect may pertain to trait resilience.

Resilience can be defined as the capability to recover from or adapt to adverse and traumatic events or major changes in the life course (Masten & Reed, 2002). A resilient personality is a constellation of traits, such as emotional tolerance, hardiness, and self-awareness that work together to ameliorate antagonistic outcomes of difficult circumstances (Skodol et al., 2010). In a study on the protective factors of positive treatment outcomes, evidence suggests that resilience was a significant predictor of recovery, concurrently with a decline in the severity of post-traumatic stress disorder symptoms (Dutton & Greene, 2010; Kunst et al., 2011). This may be applicable to other contextual outcomes, such as recovery from exposure to violence. In a study exploring the development of post-traumatic stress disorder symptoms following the subjection to a terrorizing incident, resilience was found to be a significant moderator to debilitate this association (Salami, 2010). Another study on protective factors and resilient functioning after violence exposure suggests an inverse association between the development of depression and resilience (Howell & Miller-Graff, 2014). This finding suggests that possessing trait resilience may counterbalance psychological distress, which would otherwise arise from a threatening affair. Since the literature provides evidence that violent incidents may have increased within the walls of forensic psychiatric facilities since the lockdown, and that this diminishes the general well-being of the workers, resiliency should be investigated as a possible protective factor.

Beyond the trait of resilience, forensic workers may require a certain mentality, or coping strategy, to subsist the expected amplification of aggressive behavior from inpatients. Coping is defined as the way individuals respond to and tackle struggles or calamities (Tunks & Bellissimo, 1988). According to Folkman and Lazarus (1984), the efficacy of a coping strategy is contingent on the pertinence of adjustment to internal and external factors of the situation. There are adaptive and maladaptive responses to certain stimuli, such as using adversity to foster growth, or letting unfavorable consequences overhaul standard functioning. Several facets of coping can be discerned; active coping, passive coping, and avoidant coping.

Active coping, or problem-focused coping, regards the ability to manage stressors through suitably targeted behavior. This strategy has behavioral manifestations, such as attempting to change or eradicate the stressor to diminish its impact, which ultimately expedites favorable consequences, such as less distress and enhancement of psychological well-being (Chao, 2011). Reconceptualizing the problem to find solutions could include active problem-solving or positive reappraisal of the given situation (Smith et al., 2016). A previous study investigated the correlates of trauma and coping style and how inclined victims of physical trauma and impoverishment would be in seeking mental-health support (Rayburn et al., 2005). Findings revealed that an active coping strategy predicted the greatest likelihood of seeking mental health services in traumatized victims, ultimately paving the way for psychological healing. Another study on generalized workplace abuse revealed that active problem-focused coping can minimize the vulnerability of victimized workers to suffer from unfavorable mental health consequences (Richman et al., 2001).

Another distinguished coping strategy is passive coping. A passive coping mechanism is characterized by a degree of acceptance of the stressor due to the realization that there may not be an opportunity to change, alter or discard it (Choi et al., 2012). Research has shown that a passive coping style may prolong depressive symptomatology, such as anhedonia and depletion syndrome, as a result of social withdrawal responses rather than seeking social support and engagement (Blazer, 2009). Another manifest behavioral outcome of passive coping mechanisms could be substance abuse, alcoholism, and smoking as an attempt to cope with the acceptance of the stressor (Fu et al., 2020). In the context of healthcare workers, a previous study has shown that a passive coping strategy could cause apprehension to file reports of misconduct, due to the underlying nature of acceptance and passivity of stressors (Jeong et al., 2016). Another study on the implications of client violence toward social workers reiterated the importance of an effective coping strategy, and that passive coping did not yield a positive outcome on the overall mental health of victimized workers, whereas an active coping strategy did (Padyab et al., 2013).

An avoidant coping style is a counterproductive defense mechanism against a stressor. Avoidant coping tries to escape the stressor, rather than tackling and facing the problem directly (Herman-Stabl et al., 1995). According to Carver et al. (1989), three aspects compose the mechanism of avoidant coping; focusing on and venting of emotions, behavioral disengagement, and mental disengagement. Research by Gomez (1998) indicated that an avoidant coping style was positively and independently associated with depression, and that individuals suffering from depressive psychopathology and behavioral disorders relied on an avoidant coping mechanism to manage distress (Ebata & Moos, 1991). It was also found that an over-reliance on avoidant coping disturbed the natural psychological recovery process that occurs after a traumatic event, and that those who had a continuous dependence on avoidance measures had the longest-lasting and most severe symptoms of posttraumatic stress (Pineles et al., 2011). Another recent study showed that avoidant coping strategies were positively associated with all facets of distress, and negatively associated with well-being (Dawson & Golijani-Moghaddam, 2020).

## The Present Study

The present study aims to investigate the effects of victimization on the general well-being of forensic workers. Trait resilience and various coping styles were explored as possible moderators of this association. With an abundance of literature suggesting unfavorable outcomes from a lack of social support for forensic patients, the possible effects of the lockdown measures will be investigated in the context of forensic psychiatric facilities. The present study hypothesizes that the prevalence rates of physical and verbal violence of forensic patients toward forensic workers have increased since the government issued lockdown, compared to having no lockdown (*H1*).

In addition, with the expected increase of inpatient violence in psychiatric facilities following the lockdown, there is also a presumed risk of victimization of forensic workers. Grounded on the foregoing literature, we assume a negative association between victimization and the general well-being of forensic workers (*H2*).

Focusing on optimizing the general well-being of forensic workers must be given concerted attention by investigating possible protective factors to buffer adverse effects of victimization. Since research has shown a negative association between victimization and the general well-being of forensic workers, it is likely that high resilience weakens this negative association. Therefore, we expect trait resilience to be a protective factor and to have a weakening effect on the negative association between victimization and the general well-being of forensic workers during the lockdown (*H3*).

Alongside resilience, the same effect is expected from an active coping strategy. We expect that an active coping strategy may have a weakening moderating effect on the negative association between victimization and the general well-being of forensic workers (*H4.a*). Unlike an active coping strategy, a passive coping strategy seems to have a contrasting effect on buffering of adverse consequences on well-being. This will be investigated under the assumption that a passive coping strategy will have a moderating effect on the association between victimization and general well-being (*H4.b*), such that forensic workers with a high passive coping style will have lower levels of well-being in situations of high levels of victimization. Likewise, an avoidant coping mechanism has also been found to have unfavorable outcomes in the process of recovery from an adverse event. Therefore, we expect an avoidant coping strategy of forensic workers to have an enhancing moderating effect on the negative association between victimization and well-being during the lockdown (*H4.c*).

## Methods

### Procedure

The present study is a subsection of a larger longitudinal study with four repeated measures, however, only the first two waves of data collection were utilized in this study. The first survey took place during the last week of June which is the time point where there is an expected increase of violent incidents due to the government-issued lockdown (*T1*). The second wave of data collection occurred during the last week of November, which is after the lockdown was lifted and is therefore the comparison point with the June data collection (*T2*). Participants were recruited directly from three forensic psychiatric centers (FPCs) in the Netherlands and Belgium, namely Fivoor, FPC Antwerp, and FPC Ghent. All staff working in the three institutions, both forensic workers who have contact with patients and support staff who are not in contact with patients, were informed by the Human Resources unit 2 weeks in advance of the impending research. To participate in the study, it was necessary to have sufficient knowledge of the Dutch language in order to be able to understand the questionnaires. In total, 418 staff members agreed to participate.

The study was administered through computer-based surveys in order to optimize the response rates of participants. Before completing a survey that included a set of validated psychosocial questionnaires, participants were asked to sign the consent form. Participation in the study was voluntary and respondents could decide to drop out at any time and discontinue further participation in the study. The survey took no more than 20 or 30 minutes to complete. One week before the second data collection, staff members were informed about general findings to optimize the response to the second survey.

For the purpose of the present study, only workers who have direct contact with patients on a regular basis were retained. Kitchen and cleaning staff were excluded under the presumption that there was not a high risk of victimization of these workers. Additionally, individuals were excluded if they had 8 hours or less of patient contact weekly.

The study had been approved by the boards of the institutions and by Fivoor’s Scientific Research Committee and registered at AsPredicted. Results of the study are reported at the group level and results cannot be traced back to the individual.

### Participants

Of a total of 418 participants, 100 workers without direct contact with patients were excluded from this study as well as additional seven workers who had more than 10% of missing data on questionnaires or less than 8 hours of patient contact weekly. This led to the final sample size of 311 (74.6% females) between ages 21 and 65 (*M* = 37.99, *SD* = 12.20) at T1. Most respondents were married or living together with a partner (58.9%). On average, they worked for about 6 years in the FPCs (*M* = 5.96, *SD* = 6.71). The reported average number of hours they spent with patients weekly ranged from 10 to 60 (*M* = 25.38, *SD* = 9.37). Verbal aggression by patients in the past 2 months was reported by 158 workers (50.8%), whereas 61 workers (19.6%) reported severe aggression by patients (i.e., both verbal and physical). At T2, there were 238 valid participants with an age ranging from 19 to 67.

### Materials

#### General well-being

General well-being was measured using the World Health Organization-5 Well-Being Index (WHO-5; Staehr, 1998). This five-item scale measures psychological and subjective well-being through items such as “I have felt cheerful and in good spirits” to “My daily life has been filled with things that interest me” (for the full questionnaire, see Table S1 in the Supplemental Material). Participants rated answers on a Likert scale ranging from 0 (not present) to 5 (constantly present). A literary review validated the methodological aspects of this questionnaire and found satisfactory validity as a screening tool and outcome measure of depression in most adapted language versions (Topp et al., 2015). Specifically, a Dutch research team validated this scale and found complacent psychometric properties with a Cronbach’s α of .82 (De Wit et al., 2007). In the present study, the internal consistency of the WHO-5 was very good with Cronbach’s α = .80 at T1 and α = .87 at T2.

#### Victimization

Accounts of victimization were characterized by the number of times a worker has had verbal or physical aggression directed toward them instigated by patients in the past 2 months. Verbal and physical aggression included accounts of threats and intimidation. Answer options were classified on a five-point Likert scale, ranging from 1 (none) to 5 (10 or more times). Participants were also asked if any major and distressing life event had occurred outside of the work environment at that time (and if yes, where this took place and the nature of the event), in order to control for any confounding variables (for the full questionnaire, see Table S2 in the Supplemental Material).

#### Resilience

The degree of resilience within forensic psychiatric workers was evaluated using the Resilience Evaluation Scale (RES; van der Meer et al., 2018). The English version of this scale is originally rated on a 7-point Likert scale, however, the Dutch version has been adapted to a 4-point Likert scale ranging from 1 (totally disagree) to 4 (totally agree). This 9-item scale aims at measuring five different facets of resilience; equanimity, perseverance, self-reliance, meaningfulness, and existential aloneness. The scale ranges from statements such as “I have confidence in myself” to “After setbacks, I can easily pick myself up” (for the full questionnaire, see Table S3 in the supplementary material). The psychometric properties of this scale were validated through promising convergent validity and internal consistency, with both versions showing a Cronbach’s α above .80. In the present study, the internal consistency of the RES scale was very good with Cronbach’s α = .85 at T1 and α = .87 at T2.

#### Coping style

Coping style was determined by the Utrecht Coping List ([Utrechtse Coping Lijst] UCL; Sanderman & Ormel, 1992), which is a 47-item well-validated questionnaire to measure different facets of coping, originally constructed in Dutch. Although the list distinguishes seven different coping styles, only three styles were used in this study (see Table S4 in the supplementary material), namely active coping (seven items; for example, “I intervene immediately and get rid of it”), passive coping (six items; e.g., “I give up and come up with nothing”), and avoidant coping (7 items; e.g., “I avoid the situation”). These coping mechanisms were measured by asking participants to rate hypothetical responses to stressors on a 4-point Likert scale. The internal consistency of this scale was reaffirmed in several English validations of this scale, with Cronbach’s α ranging between .67 and .86 in all subscales (Goossens et al., 2017). In the present study, the internal consistency of the three scales was satisfactory at T1: α = .80 (active coping), α = .65 (passive coping) and α = .68 (avoidant coping), and at T2: α = .77 (active coping), α = .72 (passive coping) and α = .70 (avoidant coping).

#### Statistical analysis

Statistical analyses were conducted using SPSS (Version 27, IBM Statistics). First, a missing value analysis was performed to withdraw participants that had 10% or more data missing on the WHO-5, RES, and UCL. Next, Little’s MCAR test (Li, 2013) indicated that the data were missing at random since *X*^2^(*df =* 1,577) = 1497.43, *p* = .924, allowing for imputation techniques and replacing the missing values with the mean of the corresponding variables. After excluding participants who had 8 hours or less of patient contact weekly, the valid sample consisted of 311 participants. Subsequently, a power analysis was conducted using G*Power 3.1.9.4 (Faul et al., 2007) to ensure that the sample size was satisfactory in order to detect an effect at the desired significance level. With a suggested medium effect size ρ= .3 by Cohen, a minimum sample size of 82 participants was required to reach sufficient power (1–β) = .80 at a two-tailed significance level of α = .05.

Moreover, the normality of the data was assessed by skewness and kurtosis analyses. According to these tests, all variables except passive coping were normally distributed with scores between −1.96 and 1.96 at T1 (George & Mallery, 2010 ). To investigate this further, a Shapiro-Wilk test of normality was conducted to statistically analyze whether the variables were statistically different from a normal distribution. This analysis indicated that variables did not differ statistically from a normal distribution, as they all showed a significance of *p* < .001. However, deviation from a normal distribution was expected, as violence is not normally distributed in the general population either. This led to the decision to use nonparametric statistics instead, to test *H1* and *H2*. Additionally, no multicollinearity issues were detected, as the variance inflation factor for all possible linear relationships fell below 3, excluding the possibility that explanatory variables are in exact linear function of each other.

To test whether the prevalence rates of overall victimization toward forensic workers differed significantly during the lockdown (T1), compared to no lockdown (T2; *H1*), a Mann–Whitney U test was used. Furthermore, Spearman’s correlation (*ρ*) was used to test the association between victimization and the general well-being of forensic workers at T1 (*H2*). Associations between all remaining variables were also tested using Spearman’s correlation. These correlations were investigated through a split gender variable to identify possible gender differences in the associations. Fisher’s r to z transformation was used to test whether comparisons could be made between male and female forensic workers. Statistical significance was determined at a level of significance (α = .05).

Furthermore, we used PROCESS macro for SPSS (Version 3.5.3; Hayes, 2021 ) to investigate the moderating role of resilience (*H3*) and different coping styles (*H4a*, *H4b*, *H4c*) on the association between victimization and the general well-being of forensic workers at T1. Four separate moderation analyses were conducted. Victimization was coded as multi-categorical by using the indicator method (no victimization, verbal victimization, verbal & physical victimization). The first moderation analysis consisted of resilience as the moderator to test *H3*. The remaining three moderation analyses tested the three coping styles (active, avoidant, passive) as moderators respectively (*H4a, H4b, H4c*). All other variables, such as age and gender, were entered as covariates in the models. A statistically significant effect was determined if *p* < .05. Additionally, the continuous variables in the moderation analyses were mean-centered.

## Results

Descriptive statistics for all variables studied from both T1 and T2 are displayed in Table 1. The mean score of the total number of violent incidents was significantly higher in Wave 1 (*M* *=* 3.76, *SD* = 1.94) than in Wave 2 (*M* = 2.85, *SD* = 1.51). Since a nonparametric test was chosen, the mean ranks of violent incidents between Wave 1 (285.14) and Wave 2 (239.10) were then compared. The Mann-Whitney U-test indicated that violent incidents were higher once the lockdown was implemented (Md = 3) compared to when the lockdown was lifted (Md = 3), *U* = 28,660, *p* < .001, *r* = .16. This finding supports *H1* stating that victimization was higher in Wave 1 than in Wave 2.

**Table 1.** Descriptive Statistics of Variables Influencing Well-Being in Forensic Workers.

	Timepoint							
	Wave 1: Lockdown				Wave 2: No Lockdown			
	*M*	*SD*	Min	Max	*M*	*SD*	Min	Max
Well-being	60.87	19.17	0	100	62.62	18.10	1	25
Resilience	27.38	4.05	9	36	27.10	4.21	0	36
Active coping	2.89	.48	1	4	2.81	0.42	1	4
Avoidant coping	1.66	.52	1	3	1.70	0.37	1	3
Passive coping	1.46	.54	1	3	1.63	0.42	1	3
Total violent Incidents	3.76	1.94	2	10	2.85	1.51	2	9

*Note.* Wave 1 data was conducted in June when the lockdown was implemented (*N* = 311), Wave 2 data was conducted in November after the lockdown was lifted (*N* *=* 238).

To test *H2*, the assumption that there is a negative association between victimization (violence) and the general well-being of forensic workers at T1, Spearman’s correlation (*ρ*) was calculated. As seen in Table 2, there was no significant association between being a victim of verbal violence on the well-being of males or females. Comparably, there was also no significant association between being a victim of overall victimization on the well-being of males or females.

**Table 2.** Spearman’s Rho Correlation Matrix Between Primary Variables Separated by Gender.

	1.	2.	3.	4.	5.	6.	7.	8.	9.
Males (*n* = 79)									
1. Well-being	–								
2. No victimization	−.04	–							
3. Verbal victimization	.04	−.58**	–						
4. Verbal and physical victimization	−.01	−.39	−.57**	–					
5. Resilience	.30**	.02	−.34**	.35**	–				
6. Active coping	.18	.07	−.21	.16	.48**	–			
7. Avoidant coping	−.03	−.16	.01	.14	−.22	.42**	–		
8. Passive Coping	−.20	.08	.47	−.01	−.28	−.34**	.31**	–	
9. Age	.08	.20	−.10	−.09	.03	.16	−.19	.23*	–
Females (*n* = 232)									
1. Well-being	–								
2. No victimization	.03	–							
3. Verbal victimization	−.01	−.71**	–						
4. Verbal and physical victimization	−.04	−.33**	−.47**	–					
5. Resilience	.36**	.21**	−.15*	−.05	–				
6. Active coping	.31**	.04	.02	−.07	.41**	—			
7. Avoidant coping	−.21**	−.01	.09	−.11	−.26**	−.23**	—		
8. Passive coping	−.39**	−.06	.02	.05	−.32**	−.31**	.33**	—	
9. Age	.08	.22**	−.10	−.13*	.25**	.25**	−.10	−.02	—

*Note. N* = 311 (males: *n* = 79, females: *n* = 232)

* *p* < .05. **p < .01

However, several other significant correlations were found, such as the weak positive association between resilience and well-being in males, and a moderate positive association in females. Additionally, all three coping styles had a significant association with well-being in females, namely a moderate positive association with active coping, a weak negative association with avoidant coping, and a moderate negative association with passive coping. Contrastingly, no significant associations were found between any coping style and well-being in males. The age of female forensic workers also had significant moderate positive associations with active coping and resilience, and a significant weak negative association with verbal and physical victimization. Age did not play as significant of a role in male forensic workers, except for the weak negative association with passive coping and avoidant coping. Resilience and active coping showed significant moderate positive associations with each other in males and females. We found a similar trend between the two maladaptive coping styles, as avoidant and passive coping also had a moderate positive association with one another in males and females.

To test whether resilience, active coping, avoidant coping and passive coping modify the association between victimization and well-being, several moderation analyses were conducted. The results showed that an active coping strategy significantly buffers the effect that verbal and physical victimization have on well-being. Table 3 shows the output of the analysis with active coping as the moderator, with the independent variable being separated into the types of victimization. In coding the variables, the “no victimization” group was used as the reference group. An active coping strategy significantly buffered the effect that verbal and physical victimization can have on well-being (*b* *=* −18.39, *t*[305] = −2.95, *p* = < .05, [95% CI: −30.64–6.13]). Additionally, active coping strategy and gender had a significant main effect on well-being, controlled for victimization and age (see Table 3). In other words, higher levels of active coping strategies and male gender were associated with higher levels of well-being. However, resilience, avoidant coping, and passive coping did not have any influence on the association between victimization and well-being.

**Table 3.** Moderation of Active Coping Between Levels of Victimization and Well-Being.

					95% CI	
	*B*	*SE*	*t*	*p*	LLCI	ULCI
Constant	65.51	6.98	9.53	<.01	52.77	80.26
Verbal victimization	0.73	2.38	0.31	.76	−3.96	5.42
Verbal and physical victimization	−2.16	3.07	−.70	.48	−8.19	3.88
Active coping	18.62	4.58	4.07	<.01	9.61	27.64
Active coping × Verbal victimization	−9.48	5.41	−1.75	.08	−20.14	1.17
Active coping × Verbal and physical victimization	−18.39	6.23	−2.95	<.01	−30.64	−6.13
Gender	−5.68	2.53	−2.24	<.05	−10.66	−.70
Age	0.04	0.09	0.46	.65	−.14	0.22

*Note. SE* = Standard Error.

## Discussion

The objective of the present study was to investigate the effects of victimization on the general well-being of forensic workers as well as possible protective and risk factors that could mitigate the expected ramifications. Examining these connections is important because forensic workers play an important role in the resocialization process of forensic inpatients. Although forensic workers are subject to a certain level of institutional victimization by forensic patients in general, the potential accelerating effects that a lockdown could have on violent behavior warranted further investigation.

The first hypothesis expected an increase in the victimization of forensic workers once the government-issued lockdown was implemented in forensic psychiatric facilities due to the Covid pandemic. The findings showed that forensic workers were significantly more likely to be victimized during a time of lockdown than during a time without an implemented lockdown. This finding is aligned with previous research and literature stating that stable social support throughout confinement could act as a buffer against recidivism and aggressive acts (Cochran, 2014). Furthermore, the Forensic Early Warning Signs of Aggression Inventory (FESAI; Fluttert et al., 2011) labels changes in daily activities and social isolation as the two highest-ranked warning signs of violence in forensic patients. Since the COVID-19 related lockdown has dramatically influenced the daily lives of forensic patients (e.g., no visitation rights, no leave modalities), this could be a crucial factor why violent incidents increased significantly. Additionally, a previous study investigating the relationship between nurse behavior and aggression in forensic psychiatric facilities found that violent patients seek higher levels of supervision and attention from forensic workers, compared to non-violent patients (Whittington & Wykes, 1994). The same study found that victimized forensic staff had made themself more available to patients, in terms of time and attention, than non-victimized forensic staff, which may also contribute to the vulnerability of being victimized. These findings provide a possible explanation for the increase in violence during the lockdown. As forensic patients were experiencing heightened isolation and detachment from friends and family, they may have compensated by seeking attention from forensic workers by acting aggressively (Whittington & Wykes, 1994).

The second hypothesis assumed that there was a negative association between the increase of victimization and the general well-being of forensic workers. Contrastingly, there were no significant associations between any level of victimization (verbal victimization, verbal & physical victimization) and general well-being. Therefore, the second hypothesis was not supported by the research. This finding contradicts previous research on the influence of victimization on well-being, such as the reduction in the emotional and physical health of psychiatric workers (Needham et al., 2005). However, several potential explanatory factors may have influenced this finding. Firstly, the WHO-5 Well-Being Index (Staehr, 1998) is a 5-item measure of current mental well-being over the last 2 weeks. Items such as “I have felt active and vigorous” and “My daily life has been filled with things that interest me” may not be aspects that could be strongly influenced by institutional victimization in forensic workers. Furthermore, Ryff (1989) suggests shortcomings in empirical measures of well-being as it is often operationalized as short-term affective well-being, which is also the case of the WHO-5 as it focuses on feelings in the past 2 weeks.

The WHO-5 underrepresents enduring life experiences, such as a sense of purpose and direction, which was an important yet marginalized indicator of well-being. Further research shows that workplace victimization has a detrimental impact on job satisfaction and motivation (Hesketh et al., 2003), which goes synchronously with a sense of purpose and direction in life. These are complexities of well-being that the WHO-5 Well-being Index fails to assess accurately, yet are still viable areas of interest for optimizing the well-being of forensic workers. Alternatively, if the inconsistency of our findings does not lie in the measure itself and victimization does not negatively affect the well-being of forensic workers, the concept of post-traumatic growth (PTG) could play a role in this association, however, not investigated in this study. Specifically, individuals who have overcome adversity, such as ongoing workplace victimization, display even higher levels of well-being after the traumatic events due to this phenomenon (Tedeschi & Calhoun, 2004). Our findings did not show a significant positive association that this could explain.

The third and fourth hypotheses focused on investigating the moderating effect of resilience and coping on the association between victimization and well-being. The moderation analysis found active coping to be a significant moderator between verbal and physical victimization and well-being. This signifies that forensic workers with an active coping style experience less of a negative shift in their well-being after being verbally and physically victimized. This is in line with previous findings that an active coping strategy may buffer the adverse effects of workplace victimization (van Den Brande et al., 2016). However, this specific association has not been widely investigated in an inherently stressful work environment such as a forensic institution. Additional previous research has found that victims of physical trauma, such as physical victimization in forensic workers, would be more likely to seek mental health support if they exhibit an active coping strategy (Rayburn et al., 2005). This finding could also contribute to the evidence found in the present study, as victimized forensic workers with an active coping strategy may have sought out professional help after their victimizing incident. Another study found a significant association between an active coping strategy and levels of subjective well-being, such as positive affect, however, this was in adolescents (Coyle & Vera, 2013). Nonetheless, the finding that an active coping strategy may alleviate the negative effects of victimization on general well-being of forensic workers is a step toward protecting the mental welfare of essential workers.

In contrast, results showed that resilience, passive coping, and avoidant coping were insignificant moderators of the association between victimization and well-being. This finding is not in line with previous research, as resilience has been found to debilitate the development of psychological distress symptoms after a terrorizing event (Howell & Miller-Graff, 2014). Justification as to why resilience did not moderate the association could be due to the construct itself. A study by Carbonell et al. (2002), discerned protective factors that promote resilience, such as self-esteem, social support, and coping skills. Coping skills translate into active coping strategies, meaning that active coping is only a small part of building resiliency. If other factors are needed to promote resilience, the dissimilarity between active coping and resilience in the same association makes more sense, despite their similar underlying properties. Furthermore, passive and avoidant coping did not seem to strengthen the negative association between any form of victimization and well-being. Although there is sufficient research to support the unfavorable outcomes of these maladaptive coping styles on well-being, there is not ample evidence that these coping strategies should have significantly interacted between victimization and general well-being.

### Limitations

The results of the present study should be considered with the timidity of several limitations. Firstly, we had no prevalence rates of victimization before the COVID-19 pandemic.

As a comparison period, we have chosen a period in which the COVID-19 measures for visits and movements of forensic patients and staff in the forensic centers were relaxed. It is possible that the decrease of victimization rates after the first lockdown was lifted could be attributed to the regain of social support and a certain level of freedom. This leads to the inquiry of whether there would have been as significant of a difference in prevalence rates had it been compared to a time before the pandemic.

Another limitation could be the use of the WHO-5 Well-Being Index measure. As previously explained, the WHO-5 does not assess a longitudinal grasp on well-being, but rather a very short-term scope and does not consider durability. The five items of the questionnaire do not fully assess quality or satisfaction with life. Additionally, the prevalence rates of victimization were gathered over the past 2 months rather than 2 weeks. This means that victimized forensic workers could have been suffering from acute distress or post-traumatic symptoms a month following the incident, yet the WHO-5 would not have captured this.

Lastly, the study was also limited by an unequal gender distribution, with three times more female than male respondents. This gender imbalance could lead to a misrepresentation of certain associations. Females tend to be more agreeable (Risse et al., 2018), which may explain the disparity in gender given that more female forensic workers volunteered in the present study.

Despite the above limitations, there are also some notable strengths. The present study contributes to filling the gap in the realm of forensic institution research. Since the COVID-19 pandemic has only recently emerged, the long-lasting effects it may have on psychiatric institutions are of value. Beyond the impact of a lockdown, the present study also provided further insight into possible protective factors of victimized forensic workers. Since forensic settings are often disregarded in empirical research, the well-being and safety of forensic workers have not been prioritized.

Another strength is that the valid sample size (*N* = 311) was exceptionally larger than the minimum requirement according to the power analysis (*N* > 84). This afforded us the freedom to exclude forensic workers that did not have sufficient hours of patient contact and were therefore at low risk of victimization.

### Clinical Implications

The findings of the present study may have important implications for clinical forensic practices. As existing research on the effects of the COVID-19 pandemic is still scarce, the findings of the present study can be used to optimize the safety and well-being of professionals. That said, the present study highlighted the importance of training forensic workers to have an active coping strategy, which consists of problem-solving and positive reappraisal. There may also be potential to provide a sense of social support for forensic patients who are vulnerable to aggression if socially isolated. The findings that an active coping strategy and resilience are positively associated with well-being are also crucial for developing sustainable and resilient forensic workers. According to the present study’s findings, young women are at the highest risk of being verbally and physically victimized. This may mean that this young professional group of forensic workers should be made aware of the risks of victimization and, from a preventive point of view, should be trained in communication and treatment styles to reduce the chance of victimization.

### Future Research

Replication of the present study should broaden the exploration of protective and risk factors for forensic workers. Future research should emphasize gender differences of forensic workers, as well as offenders, in the context of victimization and general well-being to gain more insight into offender-victim dynamics. This requires forensic institutions to be involved in the treatment of victimized individuals. Future studies should also consider another operationalization than the WHO-5. A possible consideration would be to measure the effects of victimization on life satisfaction instead of well-being. Life satisfaction includes facets of self-concept, personal goals, self-perceived ability to cope, and job satisfaction (Schultz, 1975). Other positive psychology-related constructs should be explored in this context, such as the influence of gratitude, mindfulness, or emotional intelligence on the association between victimization and well-being. As previously mentioned, there are also specific inventories that aim at measuring post-traumatic growth, which could be a potential area of interest for future research. Due to similar violent populations, this study may be repeated in prisons as well to optimize the well-being of prison staff.

## Conclusion

There is a shortcoming of research on forensic populations, both workers and patients, and the present study aims to minimize this gap. Overall, these findings could lead to both policy and practical implications. The well-being and safety of forensic workers can be improved with active coping training, emotional support systems, and support outlets for socially isolated forensic patients. To a practical degree, the current research provides insight into protective factors for potential victims and gender differences within. Forensic populations receive little awareness in the media and empirical research. However, the COVID-19 pandemic is having a similar impact and exasperations on both the forensic and general population, highlighting the shared humanistic response to adverse incarceration.

## Supplemental Material

sj-docx-1-ijo-10.1177_0306624X221124834 – Supplemental material for The Moderating Roles of Resilience and Coping Strategy on Well-Being of Victimized Forensic Workers Supplemental material, sj-docx-1-ijo-10.1177_0306624X221124834 for The Moderating Roles of Resilience and Coping Strategy on Well-Being of Victimized Forensic Workers by Caroline Rou, Marija Janković and Stefan Bogaerts in International Journal of Offender Therapy and Comparative Criminology
