# Supplementary material for: The Moderating Roles of Resilience and Coping Strategy on Well-Being of Victimized Forensic Workers
Source: Int J Offender Ther Comp Criminol. 2022 Sep 30;69(1):23–42. doi: 10.1177/0306624X221124834 (PMC11610200; doi:10.1177/0306624X221124834)
Supplement: sj-docx-1-ijo-10.1177_0306624X221124834 – Supplemental material for The Moderating Roles of Resilience and Coping Strategy on Well-Being of Victimized Forensic Workers [file sj-docx-1-ijo-10.1177_0306624X221124834.docx]

**Supplementary Materials**

**Supplementary Tables**

**Table S1**

*World Health Organization-5 Well-Being Index (WHO-5)- Dutch Version*

| 1. Ik voelde me vrolijk en in een opperbeste stemming [I have felt cheerful and in good spirits]. |
| --- |
| 1. Ik voelde me rustig en ontspannen [I have felt calm and relaxed]. |
| 1. Ik voelde me actief en doelbewust [I have felt active and vigorous]. |
| 1. Ik voelde me fris en uitgerust wanneer ik wakker werd [I woke up feeling fresh and rested]. |
| 1. Mijn dagelijkse leven was gevuld met dingen die me interesseren [My daily life has been filled with things that interest me]. |

**Table S2**

*Questionnaire Regarding Incidents*

| 1. Hoe vaak bent u in de afgelopen twee maanden in conflictsituaties met cliënten/patiënten betrokken geweest tijdens uw werkzaamheden, waarbij verbaal geweld (bijvoorbeeld bedreiging of intimidatie) werd gebruikt [In the past two months, how often have you been involved in conflict situations with clients/patients during your work, where verbal abuse (e.g., threats or intimidation) was used]? |
| --- |
| 1. Hoe vaak bent u in de afgelopen twee maanden in conflictsituaties met cliënten/patiënten betrokken geweest tijdens uw werkzaamheden, waarbij fysiek geweld (bijvoorbeeld bedreiging of intimidatie) werd gebruikt [In the past two months, how often have you been involved in conflict situations with clients/patients during your work, where physical violence (e.g., threats or intimidation) was used]? |
| 1. Heeft u in de afgelopen twee maanden buiten de werksituatie om een schokkende gebeurtenis meegemaakt [Have you experienced a shocking event outside of your work situation in the past two months]? |
| 1. Deze schokkende gebeurtenis vond plaats [This shocking event happened]. |
| 1. Deze schokkende gebeurtenis had te maken met [This shocking had to do with]. |

**Table S3**

*Resilience Evaluation Scale (RES)- Dutch Version*

| 1. Ik heb vertrouwen in mijzelf [I have confidence in myself]. |
| --- |
| 1. Ik kan me in een moeilijke situatie makkelijk aanpassen [I can easily adjust in a difficult situation]. |
| 1. Ik heb doorzettingsvermogen [I am able to persevere]. |
| 1. Ik kan na tegenslagen de draad weer makkelijk oppakken [After setbacks, I can easily pick up where I left off]. |
| 1. Ik ben veerkrachtig [I am resilient]. |
| 1. Ik kan goed omgaan met onverwachte problemen [I can cope well with unexpected problems]. |
| 1. Ik waardeer mijzelf [I appreciate myself]. |
| 1. Ik kan veel tegelijkertijd aan [I can handle a lot at the same time]. |
| 1. Ik geloof in mijzelf [I believe in myself]. |

**Table S4**

*Utrecht Coping List - Dutch Version*

| Active coping:   1. Grijp ik direct in en help het uit de wereld [I intervene immediately and get rid of it] 2. Zie ik dat als een uitdaging [Do I see that as a challenge] 3. Ga ik ermee aan de slag door het van alle kanten te bekijken [Do I get started by looking at it from all sides] 4. Houd ik m’n hoofd koel en los het op [I keep my cool and solve it] 5. Bedenk ik verschillende oplossingen [I come up with different solutions] 6. Ga ik het doelgericht oplossen [Am I going to solve it purposefully] 7. Zet ik eerst alle zaken op een rij [Let me first list all things] |
| --- |
| Passive coping:   1. Wil ik even niemand zien [I don't want to see anyone for a while] 2. Geef ik het op en kom tot niets [I give up and come to nothing] 3. Ga ik piekeren over het verleden [Am I going to worry about the past] 4. Vlucht ik weg in dagdromen [I flee into daydreams] 5. Laat ik me geheel en al in beslag nemen door problemen [I am completely absorbed in problems] 6. Voel ik me als verlamd [Do I feel paralyzed] |
| Avoidant coping:   1. Geef ik toe om problemen te vermijden [I give in to avoid trouble] 2. Accepteer ik de gang van zaken, ook al baal ik ervan [I accept the way things are going, even if I hate it] 3. Kijk ik de kat uit de boom [Do I see the cat out of the tree] 4. En ik vind het te moeilijk, dan ga ik het uit de weg [And I find it too difficult, then I avoid it] 5. Probeer ik me te onttrekken aan de situatie [Do I try to withdraw from the situation] 6. Laat ik de boel de boel [Let me mess things up] 7. Laat ik de zaak op z’n beloop [I'll let the matter run its course] |
